# Identification of changes in the microflora composition of Japanese horse mackerel (*Trachurus japonicus*) during storage to identify specific spoilageorganisms

**DOI:** 10.1016/j.crfs.2022.07.015

**Published:** 2022-08-10

**Authors:** Daisuke Kyoui, Yuri Fukasawa, Waka Miyanaga, Yui Nakamura, Tsutomu Yamane, Kazuki Sugita, Shun Yamadera, Marie Kai, Kai Shinoda, Taketo Kawarai, Hirokazu Ogihara

**Affiliations:** Laboratory of Food Hygiene, Faculty of Food Bioscience and Biotechnology, College of Bioresource Sciences, Nihon University, 1866 Kameino, Fujisawa City, Kanagawa, Japan

**Keywords:** Japanese horse mackerel, Microflora, *Shewanella*, Spoilage, Specific spoilage organisms, SSOs, specific spoilage organisms, TVB-N, total volatile basic nitrogen, TMAO, trimethyl amine-N-oxide, TMA, trimethyl amine, ASVs, amplicon sequence variants, PCoA, principal coordinates analysis, UPGMA, unweighted pair group method with arithmetic mean

## Abstract

Japanese horse mackerel (*Trachurus japonicus*) is an important marine resource, and its loss and waste should be reduced. This study aimed to identify the changes in the microflora composition during storage and specific spoilage organisms (SSOs) in Japanese horse mackerel, for spoilage prevention. They were stored at either 20 °C or 4 °C aerobically, and the bacterial viable counts, concentration of total volatile basic nitrogen (TVB-N), and microflora composition for each group were analyzed. Samples stored at 20 °C for 48 h showed similar viable counts to those stored at 4 °C for 168 h; however, the TVB-N concentrations increased at 20 °C, but not at 4 °C. 16S rRNA metagenome analysis showed that *Shewanella* became dominant genus in the microflora regardless of the storage temperature. However, dominant amplicon sequence variants (ASVs), which are a more detailed classification level than the genus, differed depending on the storage temperatures; therefore, dominant ASVs at 20 °C were assumed to be potential SSOs. *Shewanella* sp. Strain NFH-SH190041, which was genetically closely related to the dominant ASVs at 20 °C, was isolated, and its spoilage ability was verified. The strain NFH-SH190041 may be considered a novel SSO of Japanese horse mackerel because its 16S rRNA sequence is clearly different from those of known species.

## Highlights

• Changes in microflora in every part of the Japanese horse mackerel were visualized.
• NFH-SH190041 was assumed as novel specific spoilage organism.
• The spoilage ability of NFH-SH190041 was verified.

## Graphical abstract

**Figure:**
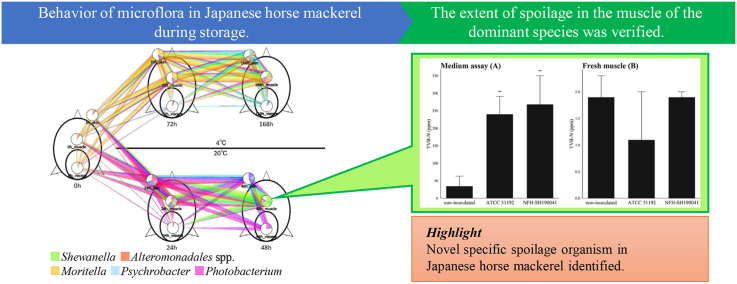

## 1 Introduction

Japanese horse mackerel (*Trachurus japonicus*), which inhabits marine water bodies around Japan and the East China Sea, is an important global marine resource, particularly in Japan, Korea, and Taiwan (FAO, 2020; Sassa et al., 2006). However, its population is reducing in comparison to those of other *Trachurus* spp. (FAO2020). In Japan, catches of *Trachurus* spp. Have reduced by 40% in the decade (Ministry of Agriculture, Forestry and Fisheries, Japan, 2021). Under these circumstances, to reduce wasteful catches and save marine resources, it is necessary to prevent food loss and waste caused by spoilage (FAO, 2020).

Some microorganisms are responsible for spoilage and are termed specific spoilage organisms (SSOs; Gram and Dalgaard, 2002; Gram and Huss, 1996). SSOs produce spoilage metabolites such as volatile amines, biogenic amines, alcohols, sulfides, and aldehydes, which are substances producing an off-odor and off-flavor (Prabhakar et al., 2020; Prester 2011). In particular, total volatile basic nitrogen (TVB-N), a general term for odor compounds, including ammonia, dimethylamine, and trimethylamine (TMA), is used as an indicator of spoilage (Ricque-Marie et al., 1998). Only few studies have reported on microorgnisms and spoilage compounds in Japanese horse mackerel. Kaneko (1970) reported that *Vibrio* spp. and *Pseudomonas* spp. were the dominant microorganisms in Japanese horse mackerel stored at 7 °C. Moreover, Kuda et al. (2002) investigated aerobic counts, TVB-N concentrations, and acid or alkaline phosphatase activity as spoilage indicators in Japanese horse mackerel stored at 4 °C. However, adequate information about the spoilage ability of microbes in Japanese horse mackerel is unavailable, and its SSOs remain unknown.

Most of the bacterial species in nature are unculturable with standard techniques (Amann et al., 1995). Likewise, it has been estimated that some bacterial members inhabiting seafoods may have been overlooked owing to their culturability (Odeyemi et al., 2018a; Zhuang et al., 2021). Based on this finding, the use of culture-independent approaches, such as molecular biological methods, represented by 16S rRNA metagenome analysis, has aided investigations on food microflora (Ercolini, 2013). 16S rRNA metagenome analysis is a comprehensive analysis in which 16S rRNA genes in the sample are sequenced by high-throughput sequencing technology, and can reveal a composition of microflora including both culturable and unculturable bacteria. Seafood such as cod, red drum, seabream, sea bass, tuna, salmon, shrimp, hybrid grouper and abalone have been also studied using this approach, and the findings provide insights on the changes in the microflora composition during storage under various conditions (Huang and Xie, 2020; Jääskeläinen et al., 2019; Kuuliala et al., 2018; Li et al., 2018; Odeyemi et al., 2018b; Parlapani et al., 2013, 2018, 2020; Remenant et al., 92 2015; Silbande et al., 2018; Syropoulou et al., 2021). This approach might provide the essentail information about the SSOs of Japanese horse mackerel, but had not been conducted yet.

The aim of this study was to monitor the microbiota composition changes in the Japanese horse mackerel during storage and to identify the SSOs. To achieve our goals, the fish were stored at 20 °C for 24 and 48 h or 4 °C for 72 and 168 h. Then, their skin, muscle, and viscera were examined to determine the microbial transfer and spoilage process. For each part, the bacterial viable counts and TVB-N concentration were measured, and the microflora composition was assessed using 16S rRNA metagenome analysis. The results were compared to identify the estimated SSOs. Finally, the isolates that were genetically closely related to the estimated SSOs were examined for trimethyl amine-N-oxide (TMAO)-reduction ability, an indicator of spoilage. The insights provided by this study will be a basic information to cralify the target for spoilage prevention.

## 2 Material and methods

### 2.1 Fish provision, storage conditions, and preparation

Round raw Japanese horse mackerel were purchased between April 2017 and November 2018 from three supermarkets in the Kanagawa Prefecture, Japan. The fish had been packed on a plastic tray covered by polyetiren film without vacuuming when sold. After purchased, the packed fish were placed on ice in a box and trasnferred to the laboratory for analysis within an hour. Forty-two Japanese horse mackerel, which were 18.0–23.7 cm (median 21.4 cm) in length and 119.1–260.6 g (median 163.6 g) in weight, were used for examination. Each was packaged into an individual plastic bag without vacuuming and stored for 24/48 h at 20 °C or 72/168 h at 4 °C. For each sampling point, six fish were provided independently. After storage, the fish was partially frozen to prevent cross-contamination of body fluids between parts. Later, a round slice between the pectoral fin and anus was collected, then the slice was separted to parts of skin, muscle, and viscera. Each part was diluted with nine volumes of buffered saline and homogenized. This suspension was used for measuring the bacterial viable counts and TVB-N concentrations and performing 16S rRNA metagenome analyses.

### 2.2 Total bacterial viable counts

The suspension prepared as described in section 2.1 was ten-fold serially diluted using 0.85% NaCl-buffered saline. The dilutions were then spread onto Marine Agar 2216 plates (Becton, Dickinson and Company, Ltd., New Jersey, USA). The plates were incubated at 20 °C for 48 h, and the colonies were counted.

### 2.3 TVB-N measurement

TVB-N was measured using partially modified microdiffusion analysis (Conway, 1952). Briefly, 1 mL of 5 mM H_2_SO_4_ was poured into the inner chamber of a Conway unit. The suspension (described above) was diluted to five volumes and poured into the outer chamber. Then, 1 mL of saturated K_2_SO_3_ was added to the outer chamber, and the lid of the Conway unit was closed immediately. The mixture was incubated at 37 °C for 1 h. After incubation, H_2_SO_4_ from the inner chamber was recovered and neutralized with 0.01 M NaOH using methyl red-methylene blue ethanol solution (Fujifilm Wako Pure Chemical Corporation, Osaka, Japan) as a pH indicator. Distilled water was used as a control. The TVB-N concentration was calculated using the following formula:

$$TVB\;-\;N\;\left(\text{ppm}\right)\;=\;0.14007\times \frac{\left(b-a\right)}{\text{amount }\;\text{of}\;\text{sample}}\;\times \;1000$$

where a is the titer of NaOH for the sample (in mL), b is the titer of NaOH for the control (in mL).

### 2.4 16S rRNA metagenome analysis

Total DNA was extracted from 1 mL of the suspension using the DNeasy Blood & Tissue Kit (Qiagen, Hilden, Germany) according to the manufacturer's instructions. The 16S rRNA V1–V2 region was amplified by PCR (Kim and Park, 2014). DNA sequencing was conducted using the Nextra Index Kit V2 (Illumina, Inc., San Diego, CA, USA) and MiSeq Reagent Kit v2 (500 cycles) (Illumina) on the MiSeq platform (Illumina). The sequence data were submitted to the DNA Data Bank of Japan under the BioProject accession number PRJDB11920.

The sequencing data were processed by the Qiime 2 pipeline using the SILVA128 SSU database (Bolyen et al., 2019; Quast et al., 2013; Yilmaz et al., 2014). First, the read sequences were clustered into amplicon sequence variants (ASVs) by DADA2 (Callahan et al., 2017) and identified taxonomically. Samples were then classified into three clusters by principal coordinates analysis (PCoA) and beta-diversity analysis. To identify the genera whose abundance differed significantly between the clusters, the abundance of each genus was compared between the clusters by one-way ANOVA (*p* < 0.05). Finally, the abundance of the ASVs based on storage time and occurrence in specific fish body parts was illustrated using TOMVi (https://doi.org/10.1101/506220).

### *2.5* *Isolation of Shewanella* sp*.*

The strains corresponding to the dominant ASVs at 20 °C were isolated. The source of isolation was the muscle tissues of the Japanese horse mackerel stored at 20 °C for 48 h, as described in section 2.1. As the medium conditions, Marine Broth 2216 (BD) supplemented either of defibrinated sheep blood (Cosmo Bio Co., Ltd., Tokyo, Japan) (50 mL/L), ammonium sulfate (Fujifilm Wako Pure Chemical Corporation) (10 g/L), yeast extract (BD) (1 g/L), or fish extract (Kyokuto Pharmaceutical Industrial Co., Ltd., Tokyo, Japan) (20 ml/L) was used. The atmosphere conditions were modified to aerobic, microaerophilic (5%–12% O_2,_ 5%–8% CO_2_; AnaeroPack-MicroAero; MITSUBISHI GAS CHEMICAL CO., INC., Tokyo, Japan), or anaerobic (<0.1% O_2_, >16% CO_2_; AnaeroPack-Anaero; MITSUBISHI GAS CHEMICAL). These medium and atmosphere conditions were combined, then total 17 culture conditions were attempted for the isolation. The sample suspension was two-fold serially diluted, used to inoculate the medium, and incubated at 20 °C for 7 days. The most diluted bacterial culture among the cultures that showed bacterial growth was subjected to 16S rRNA sequencing analysis using the Sanger method (Monciardini et al., 2002). The sequence was then aligned to the sequence of the dominant ASVs using Clustal W (Thompson et al., 1994).

### 2.6 Measurement of TVB-N concentration for estimating the spoilage ability of Shewanella sp.

NFH-SH190041 was examined for its spoilage ability. *Shewanella algae* ATCC 51192 was used as a control. Marine Broth 2216 (BD) medium supplemented with fish extract (20 mg/L) and 20 mM TMAO (Fujifilm Wako Pure Chemical Corporation) was used. Each isolate was precultured in the medium at 20 °C for 24 h and washed with buffered saline, and the OD_600_ was adjusted to 0.3. Then, 1 mL of the OD-adjusted suspension was inoculated into the 10 mL of the fresh medium or 10 g of fresh muscle tissue of Japanese horse mackerel and incubated at 20 °C for 24 h. After incubation, 1 mL of the medium was subjected to TVB-N concentration mesurement instead of the suspention described in section 2.3. Moreover, the fresh muscles were homoginized as described in section 2.1, and their suspension subjected to TVB-N concentration mesurement.

## 3 Results

### 3.1 Changes in bacterial viable counts and TVB-N concentrations

**Fig. 1:**
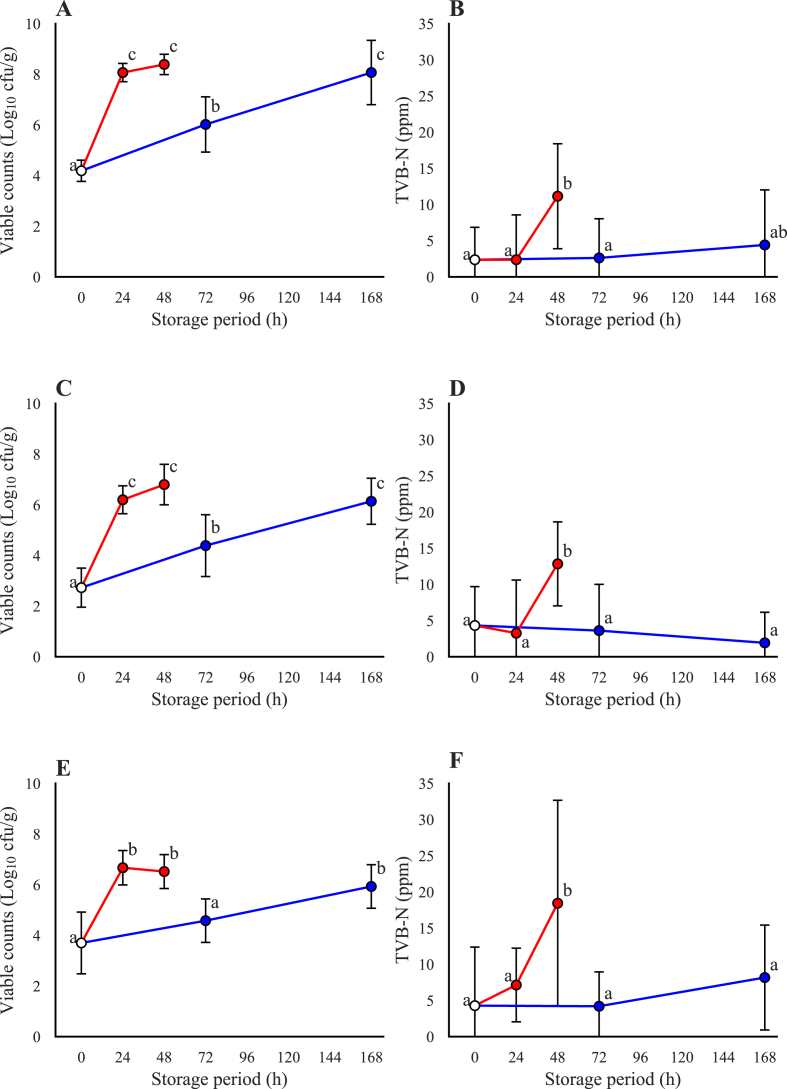
Trends of changes in bacterial viable counts and total volatile basic nitrogen (TVB-N) concentrations during storage of fish at 20 °C and 4 °C. Red, 20 °C; blue, 4 °C; white, before storage. A and B: skin; C and D: muscle; E and F: viscera. The letters near the plot indicate significance calculated by one-way ANOVA (*p* < 0.05). (For interpretation of the references to color in this figure legend, the reader is referred to the Web version of this article.)

The viable counts increased rapidly in all samples of the Japanese horse mackerel after 24 h of storage at 20 °C. In contrast, the viable counts increased gradually when the samples were stored at 4 °C for 168 h, the counts were similar to those in samples stored at 20 °C for 48 h (Fig. 1A, C, E). The storage temperature affected the TVB-N concentrations post-storage (Fig. 1B, D, F). The TVB-N concentrations increased drastically between 24 and 48 h with storage at 20 °C, whereas they remained almost unchanged with 168 h of storage at 4 °C.

### 3.2 PCoA and clustering of samples

**Fig. 2:**
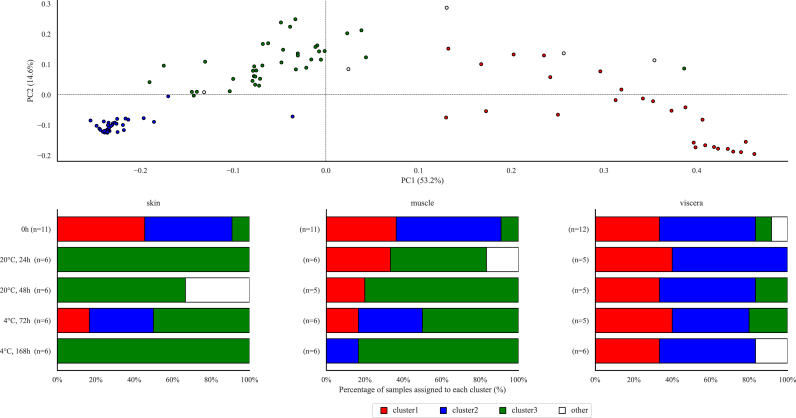
Principal coordinates analysis (PcoA) based on microflora composition. The upper part shows the PCoA plots. The lower part shows the cluster assigned for each sample condition.

The microflora isolated from the skin, muscle, and viscera of the fish in each storage period was compared by PCoA based on the ASV composition percentages and roughly classified into three clusters (Fig. 2). In addition, the storage conditions of the samples from each cluster were summarized. The microflora assigned to clusters 1 and 2 was assumed as “fresh microflora” because most of the samples assinged to these clusters were collected before storage. In contrast, the microflora assigned to cluster 3 was assumed as “spoiled microflora” because most of the skin and muscle samples belonging to this cluster were collected after storage. Most viscera samples were assigned to cluster 1 or 2, and the assigned cluster remained unchanged through the storage period.

### 3.3 Dominant genera in post-spoilage microflora

**Fig. 3:**
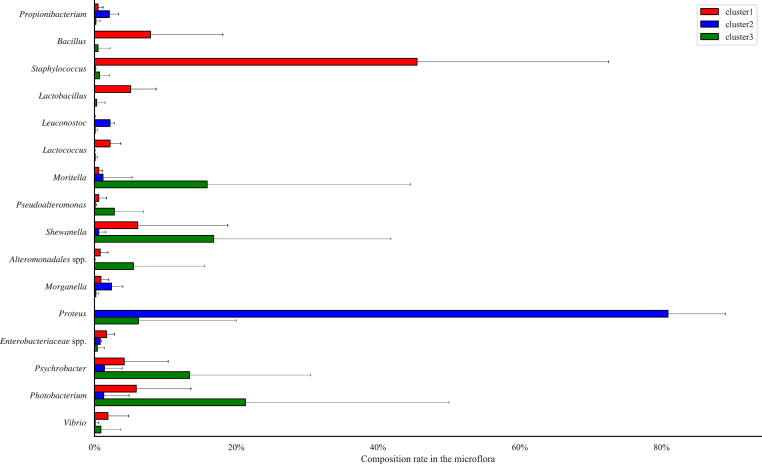
Abundance of the genera in each cluster. The genera with significantly different abundances among the clusters are shown.

To determine the genera associated with spoiled mackerel, the microflora compositions at the genera level were compared by one-way ANOVA between the clusters. For 16 genera, the abundance was significantly different between the clusters (Fig. 3). In cluster 1, which was assumed to include fresh microflora, the abundance of gram-positive bacteria, such as *Bacillus*, *Staphylococcus*, and *Lactobacillus*, was higher than that in the other clusters. The abundance of members of the *Enterobacteriaceae* family, including *Morganella* and *Proteus*, was higher in cluster 2, which was also assumed to include fresh microflora, than in other clusters. In cluster 3, which was assumed to include spoiled microflora, the abundance of marine bacteria, such as *Alteromonadales* spp., *Moritella*, *Shewanella*, *Psychrobacter*, and *Photobacterium*, was higher than that in other clusters.

### 3.4 Behavior of ASVs assigned to the dominant genus in cluster 3

**Fig. 4:**
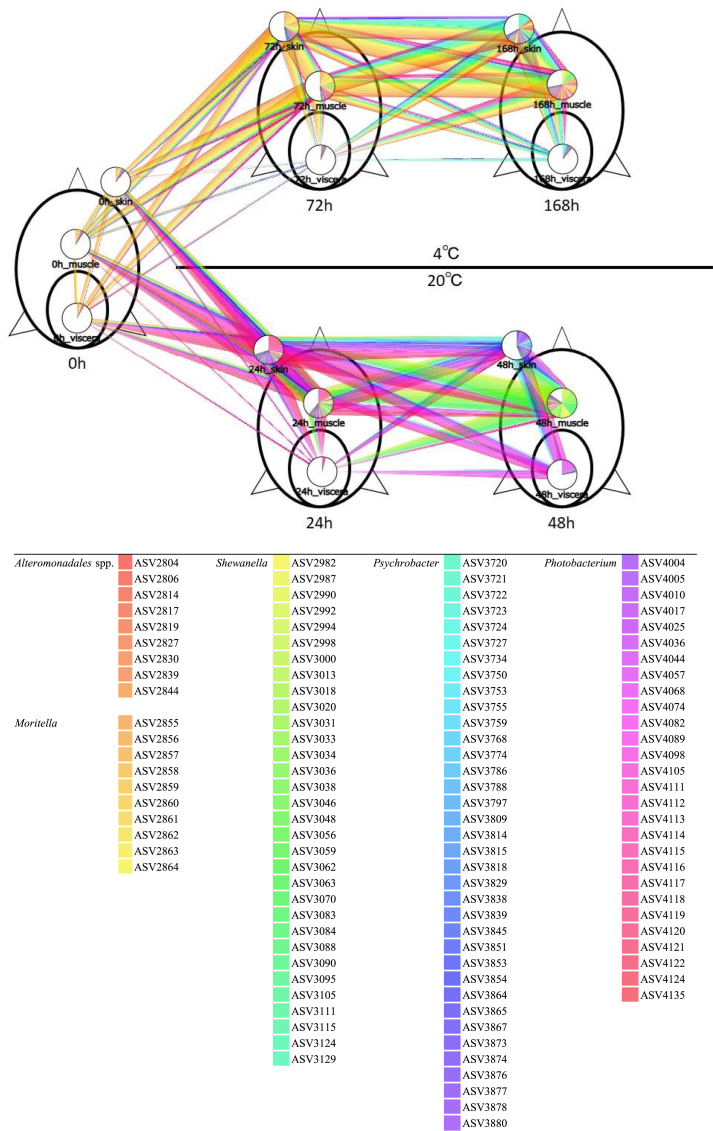
Abundance of amplicon sequence variants (ASVs) assigned to the dominant genus in spoiled Japanese horse mackerel. The plot that indicates the rate of each ASV as a circle graph. The plots that were connected by lines colored to correspond to the ASVs and their thickness indicate abundance.

For a detailed comparison of the microflora, the changes of composition of ASVs assigned to the dominant genus in cluster 3 was illustrated (Fig. 4). The observations revealed that different ASVs were dominant based on the storage temperature. In particular, in muscle samples, ASV3000, ASV3031, ASV3034, ASV3070, and ASV3083 were dominant with storage at 20 °C, whereas ASV2982, ASV3020, ASV3095, and ASV3038 were dominant with storage at 4 °C. These dominant ASVs at 20 °C and 4 °C were assigned to the genus *Shewanella*. Therefore, the genus *Shewanella* was dominant regardless of the storage temperature. However, because different ASVs were dominant depending on the storage temperature, it was considered that the different species or strains of *Shewanella* became dominant corresponding to the strorage temeperature.

### 3.5 Spoilage ability of NFH-SH190041

**Fig. 5:**
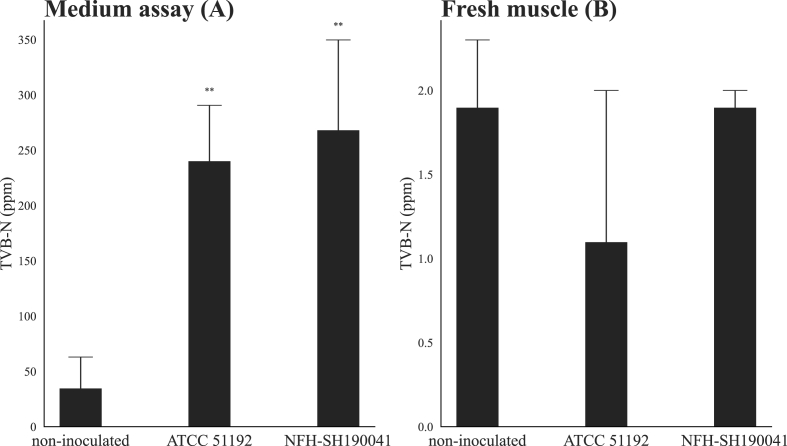
Total volatile basic nitrogen (TVB-N) produced in the medium and fresh muscle tissues of Japanese horse mackerel. A; medium, B; fresh muscle tissues of Japanese horse mackerel. TVB-N production by both *Shewanella algae* ATCC 51192 and NFH-SH190041 was compared to that in the non-inoculated condition by one-way ANOVA using Dunnett's test.

To verify the spoilage ability of the ASVs that were dominant in the muscle samples stored at 20 °C (ASV3000, ASV3031, ASV3034, ASV3070, and ASV3083), we attempted to isolate live bacteria. First, the V1–V2 16S rRNA sequences of the dominant ASVs were collated with the NCBI database using online BLAST (Madden et al., 1996), which revealed that the sequences of the ASVs were clearly different from those of known species (most related species were *S. algae*, with 93.45% identity; Supplemental File 1). Isolation was attempted using the procedure commonly used for *Shewanella* spp. (Whitman et al., 2015); however, the isolates corresponding to the ASVs could not be obtained (data not shown). Then, total 17 culture conditions with limiting dilution method were enployed, and 233 isolates obtained. Of these isolates, the strain NFH-SH190041 showed a 16S rRNA sequence closely related to those of the dominant ASVs. In addition, the strain was isolated using Marine Broth 2216 supplemented with fish extract (20 ml/L) under microaerophilic conditions. The spoilage ability of NFH-SH190041 was then verified based on its ability to reduce TMAO in the medium and thereby produce TVB-N in the freshly isolated muscle tissues of Japanese horse mackerel (Fig. 5). NFH-SH190041 exhibited TMAO reduction ability in the medium; however, it did not produce TVB-N in the fresh muscle tissue.

## 4 Discussion

### 4.1 Spoilage levels of Japanese horse mackerel

Microbial viable counts and TVB-N concentrations are common indicators of the freshness of seafood. In this study, the viable counts reached the same levels at the end of the storage period regardless of the storage temperature. These viable counts indicated that the fish were “spoiled” (Kuda et al., 2002). On the other hand, TVB-N concenterations did not increase significantly when the fish were stored at low temperature. Based on these results, it was considered that the microflora composition or the metabolic activity could differ depending on the storage temperature.

Even though the TVB-N concentration was low after storage when stored at low temeperature, the fish seemed to be spoiled as evaluated through sensory perception. It was assumed that spoilage of organic compounds other than TVB-N, such as biogenic amines, alcohol, and aldehydes, which can cause an off-odor and off-flavor, may have been produced in the Japanese horse mackerel at low temperatures (Kuuliala et al., 2018; Silbande et al., 2018; Zhuang et al., 2021). Therefore, evaluation criteria other than the TVB-N concentration may be necessary to identify SSOs at low temperatures (Prabhakar et al., 2020).

### 4.2 Changes in microflora compositon during storge

The microflora composition of Japanese horse mackerel was determined and clustered by 16S rRNA metagenome analysis. The microflora before storage, which indicates a “fresh microflora”, was dominated by Gram-positive bacteria and some *Enterobacteriaceae* members such as *Proteus*. These genera could be correlated to land animals, including humans, and may have contaminated the fish during the handling process after the catch (Drzewiecka, 2016). On the other hand, other *Enterobacteriaceae* members such as *Morganella* are marine bacteria and may inhabitat Japanese horse mackerel. In summary, the fresh microflora comprised marine bacteria that originated from indigenous microflora and contaminating bacteria that were introduced during the handling process after the catch (Parlapani et al., 2020).

The microflora of both high- and low-temperature stored fish converged to one cluster that was assumed to represent the “spoiled microflora”. This microflora was dominated by *Alteromonadales* spp., *Moritella*, *Shewanella*, *Psychrobacter*, and *Photobacterium,* which are well known as seafood spoilage bacteria (Odeyemi et al., 2018a). The results assumed these genera may become dominant in Japanese horse mackerel during storage regardless of the storage temperature and inital abundance in the microflora, and result in spoilage.

The composition of the microflora and total viable counts converged regardless of the storage temperature, while TVB-N concentrations were affected by temperature. This disagrrement could be explained by diffrences in the composition of the microflora at the ASVs level, which is a more detailed classification level than the genus. The present study demonstrates that the dominant ASVs clearly differed between the storage temperatures, indicating that the microflora composition differed at the species or strains level, which may affect TVB-N production during storage.

### *4.3* *Spoilage ability of Shewanella* sp. *NFH-SH190041*

Because most of the dominant ASVs in the muscles were assigned to *Shewanella* when samples were stored at 20 °C, *Shewanella* sp. NFH-SH190041, as genetically closely related species, was isolated and its spoilage ability examined based on TMAO reductionabiliy. NFH-SH190041 could reduce TMAO in the medium but not produce TVB-N in fresh muscle. TMAO is an osmolyte for several marine organisms (Arata et al., 1992). Therefore, it was hypothesized that NFH-SH190041 could not take up or access the precursors of TVB-N in the fresh tissue. This hypothesis was supported by the result that the TVB-N concentrations and proportions of dominant ASVs related to NFH-SH190041 increased drastically between 24 and 48 h at 20-°C storage. Moreover, some reports suggested that degradation by other microorganisms, mediated by extracellular enzymes such as protease and lipase, could promote the spoilage activity of SSOs (Dabadé et al., 2015; Ge et al., 2017). In summary, NFH-SH190041 most likely played a role as SSO in producing volatile amines from TMAO, peptides, or amino acids, which were produced by degradation of the tissue by other microorganisms and/or autolysis. NFH-SH190041 may be a novel species of *Shewanella*, which has not been well-characterized genetically or phenotypically. Moreover, the activities of the degradation enzymes of NFH-SH190041 and other microorganisms have not been examined. Therefore, further research on NFH-SH190041 and its neighbor species is necessary to determine their roles in seafood spoilage.

This study analyzed the microflora composion for every part of Japanese horse mackerel to visualize invasion of SSOs into the tissue. Fish muscle is commonly sterile until invaded by microorganisms from the skin, gill, and intestine (Chaillou et al., 2015; Ghaly et al., 2010; Kontominas et al., 2021), and it is important to specify the origin of SSOs to prevent spoilage. Unfortunately, the origin of the dominant ASVs related to NFH-SH190041 could not be determined because they were detected at low proportions in all parts of the fish before storage. From another point of view, this study demonstrated that ASVs could dominate locally. There is limited information on the differences in microflora composition and SSO abundance between body parts (Fogarty et al., 2019; Svanevik and Lunestad, 2011), and further research on these aspects will be helpful in identifying spoilage mechanisms and developing prevention strategies to avoid the spoilage of seafoods, not only for Japanese horse mackerel but other species as well.

### 4.4 Dominant genera other than Shewanella

Besides *Shewanella*, the dominant genera in the skin and viscera were *Photobacterium* in samples stored at 20 °C and *Psychrobacter* in samples stored at 4 °C. Both genera are well known as SSOs with respect to seafood (Odeyemi et al., 2018a). It was reported that *Photobacerium phosphorium* dominated under modified atmospheric packaging containing CO_2_ owing to its ability to respire anaerobically (Gram and Huss, 1996; Kuuliala et al., 2018). Therefore, the viscera environment, assumed to be anaerobic, might be suitable for some *Photobacterium* ASVs, such as ASV4036 and ASV4098. On the other hand, other *Photobacterium* ASVs dominated on the skin of Japanese horse mackerel. Fogarty et al. (2019) reported the growth of *Photobacterium* spp. on the skin of salmon stored aerobically. Therefore, it was assumed that different species or strains of *Photobacterium* grew on each body part, corresponding to environmental factors such as breathability.

In the skin and viscera samples stored at 4 °C, the same ASVs (ASV3720 and ASV3721) dominated. It is known that *Psychrobacter* produces no or low levels of volatile organic compounds (Broekaert et al., 2013). This could possibly explain the low TVB-N concentration with storage at 4 °C. However, *Psychrobacter* is also known to produce lipase and acids from carbohydrates, which can lead to the production of off-flavors, even when TVB-N is not produced (Odeyemi et al., 2018a).

## 5 Conclusions

In the present study, the microflora composition in Japanese horse mackerel during storage was visualized, and the new SSO strain NFH-SH190041 was identified. This study demonstrated that a combination of culture-dependent and -independent approaches promotes identification of SSOs. Several SSOs in seafood may still be unidentfied, and it is expected find further SSOs, similar to NFH-SH190041 discovered in the present study. NFH-SH190041 exhibited spoilage ability, but it was only a part of the spoilage process. It was considered that further resarch about on the neighbouring speciesrs which that provide produce off-odor or -fravor precursors to SSOs is necessary for further insights into the spoilage process.

## Funding

This work was supported by a research grant from the OSIMO Foundation 2016.

## CRediT authorship contribution statement

**Daisuke Kyoui:** Conceptualization, Writing – original draft. **Yuri Fukasawa:** Investigation. **Waka Miyanaga:** Investigation. **Yui Nakamura:** Investigation. **Tsutomu Yamane:** Investigation. **Kazuki Sugita:** Investigation. **Shun Yamadera:** Investigation. **Marie Kai:** Investigation. **Kai Shinoda:** Investigation. **Taketo Kawarai:** Writing – review & editing. **Hirokazu Ogihara:** Supervision.

## Declaration of competing interest

The authors declare that they have no known competing financial interests or personal relationships that could have appeared to influence the work reported in this paper.

## Appendix A Supplementary data

Multimedia Component 1 **Supplemental File 1. Taxonomic tree showing the amplicon sequence variants (ASVs) dominant in the muscle sample stored at 20 °C, strain NFH-SH190041, and *Shewanella* type strains.** The tree was constructed using the unweighted pair group method with arithmetic mean (UPGMA) based on the sequence of the 16S rRNA V1–V2 region aligned using ClustalW. Multimedia Component 1
