# Supplementary figures and images for: Identification of changes in the microflora composition of Japanese horse mackerel (Trachurus japonicus) during storage to identify specific spoilageorganisms
Source: Curr Res Food Sci. 2022 Aug 10;5:1216–24. doi: 10.1016/j.crfs.2022.07.015 (PMC9391519; doi:10.1016/j.crfs.2022.07.015)

0.01

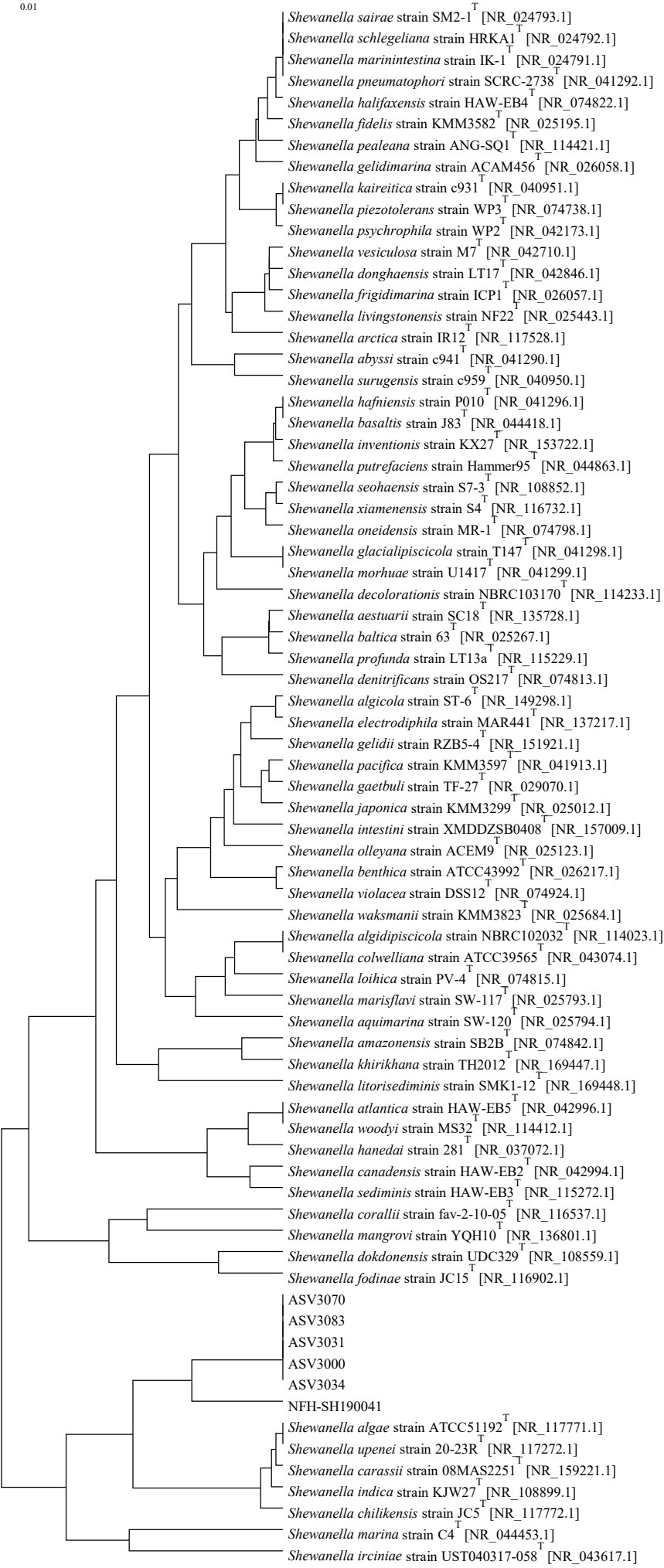

Supplement: Multimedia Component 1 — Supplemental File 1. Taxonomic tree showing the amplicon sequence variants (ASVs) dominant in the muscle sample stored at 20 °C, strain NFH-SH190041, and Shewanella type strains. The tree was constructed using the unweighted pair group method with arithmetic mean (UPGMA) based on the sequence of the 16S rRNA V1–V2 region aligned using ClustalW. [file mmc1.pdf]
